# Iron-Vanadium Incorporated Ferrocyanides as Potential Cathode Materials for Application in Sodium-Ion Batteries

**DOI:** 10.3390/mi14030521

**Published:** 2023-02-23

**Authors:** Thang Phan Nguyen, Il Tae Kim

**Affiliations:** Department of Chemical and Biological Engineering, Gachon University, Seongnam-si 13120, Gyeonggi-do, Republic of Korea

**Keywords:** sodium-ion batteries, Prussian blue, Prussian green, vanadium, high-voltage cathode

## Abstract

Sodium-ion batteries (SIBs) are potential replacements for lithium-ion batteries owing to their comparable energy density and the abundance of sodium. However, the low potential and low stability of their cathode materials have prevented their commercialization. Prussian blue analogs are ideal cathode materials for SIBs owing to the numerous diffusion channels in their 3D structure and their high potential vs. Na/Na^+^. In this study, we fabricated various Fe-V-incorporated hexacyanoferrates, which are Prussian blue analogs, via a one-step synthesis. These compounds changed their colors from blue to green to yellow with increasing amounts of incorporated V ions. The X-ray photoelectron spectroscopy spectrum revealed that V^3+^ was oxidized to V^4+^ in the cubic Prussian blue structure, which enhanced the electrochemical stability and increased the voltage platform. The vanadium ferrocyanide Prussian blue (VFPB1) electrode, which contains V^4+^ and Fe^2+^ in the Prussian blue structure, showed Na insertion/extraction potential of 3.26/3.65 V vs. Na/Na^+^. The cycling test revealed a stable capacity of ~70 mAh g^−1^ at a rate of 50 mA g^−1^ and a capacity retention of 82.5% after 100 cycles. We believe that this Fe-V-incorporated Prussian green cathode material is a promising candidate for stable and high-voltage cathodes for SIBs.

## 1. Introduction

Modern devices that harvest sustainable energy from solar light, wind, or tides and convert it to electrical energy for industrial, agricultural, and portable applications have been increasingly attracting research attention [1,2]. Rechargeable batteries are being used for the storage and management of these renewable energies to meet energy demands [3,4,5,6,7]. Lithium-ion batteries (LIBs) are the most common batteries used in mobile devices, energy storage systems, and electronic vehicles [8,9,10]. Li is the lightest metal and possesses high gravimetric energy density and high volumetric energy; therefore, LIBs can easily be integrated into portable devices such as mobile phones, smart watches, and wireless earphones [11,12,13]. However, LIBs have limited application in large-scale systems owing to their short lifetime, toxic production, and toxic recycling processes [5,12,14]. The use of LIBs also increases the device temperature and even poses the risk of explosion owing to the expansion of electrodes [15]. Sodium-ion batteries (SIBs) can be a good replacement for LIBs owing to their eco-friendliness, the safety of the chemicals used, and their relatively low cost [14,16,17,18]. Na ions have a similar working principle to that of Li ions and thus can be used for the development of cathodes, anodes, and electrolytes [19].

The development of cathode materials for metal-ion batteries is challenging because the ideal cathode material should have a high working potential, a stable voltage platform, and a high energy density [20]. Prussian blue analogs (PBAs) are ideal cathode materials for SIBs owing to their three-dimensional lattice structure, which allows the facile insertion and removal of Na ions [21,22,23]. A typical PBA compound A_x_M[Fe(CN)_6_]_y_ contains an alkali metal A (Li, Na, K) and a transition metal M (Fe, Co, Ni, Cu, V, etc.) bonded with [Fe(CN)_6_]^4−^ in a framework, creating a face-centered cubic structure (Fm3-m) [24,25,26,27]. This structure provides numerous sites for cation diffusion during electrochemical reactions [26,28]. Sodium ferrocyanide possesses the highest number of Na ions, but its high solubility limits its practical usage. Qian et al. combined Na_4_Fe(CN)_6_ with carbon to prepare a high-capacity cathode material for SIBs [29]. However, this cathode material was not stable owing to the solubility of pure sodium ferrocyanides and its low capacity at a high current rate. The insoluble PBA containing Fe^3+^ was a suitable replacement owing to the low cost, abundance, and simple processing of Fe ions. Sun et al. synthesized Fe_4_[Fe(CN)_6_]_3_, which exhibited a high sodium storage capacity of 146 mAh g^−1^ and discharge plateaus of ~3.2 V vs. Na/Na^+^ [30]. This unstable voltage platform limited its application. To overcome this issue, various metals such as Ni, Cu, Mn, and Co have been used to partially replace the Fe^3+^ ions [23,31,32]. Matsuda et al. used Mn to synthesize Na_x_Mn[Fe(CN)_6_]_y_•3.5H_2_O, which exhibited a high discharge voltage of 3.4 V vs. Na/Na^+^ [33]. Fu et al. used Ni as a metal dopant for PBA and achieved a discharge capacity of 117 mAh g^−1^ [34]. Xu et al. fabricated a binary PBA with Mn and Ni, which showed high stability [35]. Wang et al. reported that the structure of rhombohedral PBAs could enhance their electrochemical performance [36]. These results show the successful development of stable and high-capacity cathode materials. However, their voltage profiles and rate performances should be improved further.

Baster et al. recently used V(II) in PBA and observed a significant change in the voltage profiles, where the working potential was ~3.3 V vs. Na/Na^+^ [37]. However, the capacity was reduced two times compared to those of the other PBAs. Pan et al. modified V_2_O_5_ to VOC_2_O_4_ to synthesize sodium vanadium hexacyanoferrate, which showed high stability and high rate performance [38]. The presence of VO in a Prussian blue compound increased the redox potential and facilitated a stable electrochemical reaction [39,40]. However, the effects of V on PBA have not been explored thus far. In this study, vanadium ferrocyanide (VPB) was synthesized directly in a one-step solvothermal method. The combination of V and Fe in PBA was modulated by differentiating the ratios of Fe and V. Finally, the VFPB electrode, especially the VFPB1 electrode containing V^4+^ and Fe^2+^ in the Prussian blue structure, showed stable Na insertion/extraction potential of 3.26/3.65 V vs. Na/Na^+^ with reversible capacity for 100 cycles. A detailed discussion on the dynamic kinetics of the cathode materials with solid evidence from the related analysis technique was explored.

## 2. Materials and Methods

### 2.1. Chemical Materials

Iron (III) chloride (FeCl_3_, anhydrous), vanadium (III) chloride (VCl_3_), potassium ferrocyanide (K_3_Fe(CN)_6_•4H_2_O), N-methyl-2-pyrrolidone (NMP, anhydrous), sodium perchlorate (NaClO_4_), and polyvinylidene fluoride (PVDF, MW 534,000) were purchased from Sigma-Aldrich (St. Louis, MO, USA). Super-P amorphous carbon black (C, ~40 nm), absolute ethanol, ethylene carbonate (EC), propylene carbonate (PC), and fluoroethylene carbonate (FEC) were purchased from Alpha Aesar Inc. (Tewksbury, MA, USA).

### 2.2. Synthesis of Vanadium Ferrocyanide

VCl_3_ was weighed in an Ar-filled glovebox owing to its sensitivity to air humidity and water. For VPB synthesis, 0.31 g of VCl_3_ was dissolved in 15 mL of ethanol, denoted as Solution A. K_3_Fe(CN)_6_ (1.2 g) was dissolved in 100 mL of de-ionized (DI) water, denoted as Solution B. Solution A was added dropwise to Solution B while heating at 60 °C and stirring. The initially yellow-green solution turned dark green in color after 2 h, indicating reaction completion. The precipitate was washed with DI water (×2) and ethanol (×4), filtered, and freeze-dried at –80 °C for 2 d in a freeze dryer (Labconco Corp., Kansas, MO, USA).

To prepare Fe-V-mixed PBAs, Solution A was added dropwise to Solution B such that the amount of 0.1 M of FeCl_3_ was added to Solution B in amounts of approximately 1, 5, and 15 mL of FeCl_3_. Pure Fe PBA (FPB) was prepared using FeCl_3_ and Na_4_Fe(CN)_6_ instead of VCl_3_ and K_3_Fe(CN)_6_, respectively. When adding 1 mL of FeCl_3_, the precipitate was light green in color. Meanwhile, other precipitates were blue in color after adding 5 mL or more of FeCl_3_, indicating the formation of other products. Therefore, only those samples with 1 and 5 mL of FeCl_3_ were further investigated and marked as VFPB1 and VFPB2, respectively. VFPB1 was bright green in color, whereas VFPB2 was blue in color.

### 2.3. Material Characterization

The structural analysis of the samples was conducted using X-ray diffraction (XRD, D8 ADVANCE, by Bruker AXS, Ma, US; Cu Kα radiation, λ = 0.154 nm) over the 2θ range of 10–70° and transmission electron microscopy (TEM, TECNAI G2F30, FEI Corp., Hillsboro, OR, USA) at an accelerating voltage of 200 kV and in the bright field mode. The lattice spacing in a high-resolution TEM image was analyzed using Gatan microscopy suite software. The sample morphologies were also analyzed using scanning electron microscopy (SEM, Hitachi S4700, Tokyo, Japan) at an accelerating voltage of 5 kV. The elemental core levels were measured by using an X-ray photoelectron spectrometer (XPS, Kratos Analytical Ltd., Manchester, UK) under a pressure of 10^−6^ Torr. The fitting of XPS parameters was conducted by XPSpeak 4.1 software with Shirley background type.

### 2.4. Electrochemical Measurements

The electrochemical properties of SIBs were evaluated using a sodium anode in a coin half-cell assembly (CR 2032, Rotech Inc., Gwangju, Korea). The cathode was prepared by casting a slurry of 80% active materials with 10% conductive super-P carbon and 10% PVDF as the binder on aluminum foil. The electrode was dried overnight at 70 °C under vacuum. The final amount of electrode material on the cathode was approximately 1.5–2 mg cm^−2^. The glass-fiber membranes were employed as separators, and a 1 M NaClO_4_ solution in a mixture solvent of PC:EC (1:1, FEC 5%) was used as the electrolyte. The battery structures were assembled in an Ar gas-filled glovebox. The galvanostatic electrochemical charge/discharge performances of SIB cells were measured using a battery cycle tester (WBCS3000, WonAtech, Seocho-gu, Seoul) in a voltage range of 2.0–4.0 V vs. Na/Na^+^. Cyclic voltammetry (CV) tests were performed using ZIVE MP1 (WonAtech, Seocho-gu, Seoul) across a voltage range of 2.0–4.0 V. All the specific capacities were calculated based on the weights of the active materials.

## 3. Results and Discussion

**Figure 1:**
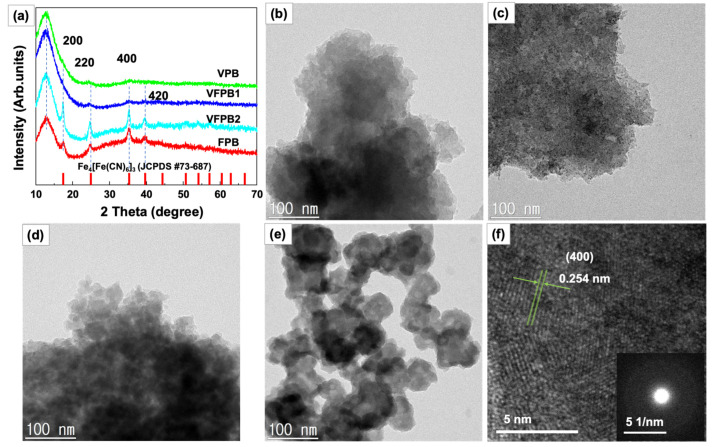
(**a**) X-ray diffraction patterns of VPB, VFPB1, VFPB2, and FPB samples; transmission electron microscope images of (**b**) VPB, (**c**) VFPB1, (**d**) VFPB2, and (**e**) FPB; (**f**) high-resolution image of VFPB1 samples; inset: selected area electron diffraction pattern.

Figure 1a shows the X-ray diffraction (XRD) patterns of VPB, VFPB1, VFPB2, and FPB. All samples exhibited low crystallinity as they were prepared at the relatively low temperature of 60 °C. The peak at 2θ = 12° indicates the stacking of PBA particles. Higher concentrations of Fe^3+^ in FPB and VFPB2 resulted in higher crystallinity and sharper XRD peaks, which are consistent with the standard peak of V_1.5_Fe(CN)_6_ (JCPDS# 00-042-1440) (lattice constant (a) = 10.13 Å) [30], whereas higher concentrations of V resulted in lower crystallinity. It is noted that the lattice constants of FeFe(CN)_6_ (JCPDS#01-0239) and Fe_4_[Fe(CN)_6_]_3_ (JCPDS#73-687) are very close to that of V_1.5_Fe(CN)_6_ (a~10.13–10.18 Å). [30,41] The widths of XRD peaks for VPB and VFPB1 were lower than those for FPB and VFPB2, indicating small crystal size.

To confirm the morphologies of each sample, scanning electron microscopy (SEM) and transmission electron microscopy (TEM) images were obtained (Figure S1 and Figure 1b–f, respectively). The aggregated particles during the drying process are shown in Figure S1. Figure 1b–e show the TEM images of samples VPB, VFPB1, VFPB2, and FPB. Crystallization at 60 °C resulted in low crystallinity and small particle sizes in the range of 10–30 nm. Only FPB containing Fe^3+^ exhibited large particles with sizes in the range of 100–200 nm. The small particle sizes of V-containing samples could be attributed to the different sizes and oxidation states of the V ions, which would result in new bonding or a slight distortion in the cubic structure of pure FPB, thus preventing the growth of the particles. In addition, the high-resolution TEM image of VFPB1 showed a lattice spacing distance of 0.254 nm, which corresponds to the (004) plane of the Prussian blue cubic structure, as shown in Figure 1f. Moreover, the selected area electron diffraction (SAED) patterns of all four samples suggest an amorphous structure, which could be attributed to the small particle size and surface defects, as shown in the inset of Figure 1f. The TEM images and SAED patterns of each sample are also shown in Figure S2.

**Figure 2:**
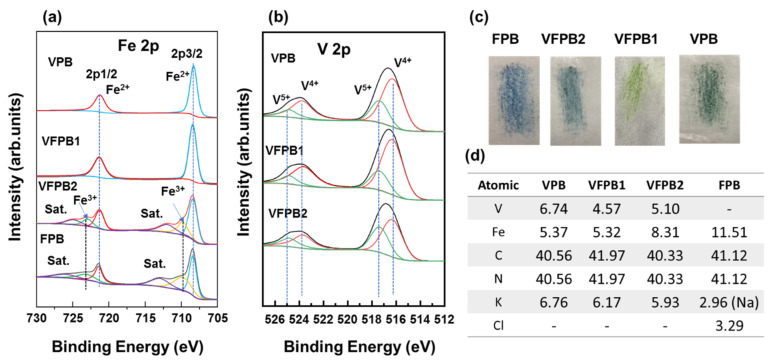
High-resolution X-ray photoelectron spectra of (**a**) Fe and (**b**) V; (**c**) photographs of Prussian blue-green sample powders on weighing paper; and (**d**) atomic percentage of V, Fe, C, N, K(Na), and Cl in VPB, VFPB1, VFPB2, and FPB.

X-ray photoelectron spectroscopy (XPS) was performed to visualize the elemental compounds and binding states of each element, especially Fe and V ions, and to further understand the structure of the Fe-V-incorporated Prussian blue samples. Figure 2a shows the high-resolution XPS spectra of the Fe 2p orbitals of VPB, VFPB1, VFPB2, and FPB. The Fe 2p peaks of VPB and VFPB1 were observed at 708.4 and 721.4 eV, which are attributed to Fe^2+^ 2p_3/2_ and 2p_1/2_. This revealed that Fe exists mainly in the Fe^2+^ state in VPB and VFPB1. Meanwhile, the Fe^3+^ from K_3_Fe(CN)_6_ is not present in these samples. The electrochemical reaction VO^2+^+2H^+^+e ↔ V^3+^+H_2_O exhibits a redox potential of ~0.337 V, whereas [Fe(CN_6_)]^3−^+e ↔ [Fe(CN_6_)]^4−^ shows a redox potential of ~0.358 V [42]. This indicates that V(III) ions have reduced Fe^3+^ in K_3_Fe(CN)_6_ to Fe^2+^, resulting in [Fe(CN_6_)]^4−^ and V^4+^ [42]. The reaction Fe^3+^+e ↔ Fe^2+^ demonstrates a redox potential of 0.771 V; therefore, low concentrations of Fe^3+^ in the PBA crystals imply high concentrations of Fe^2+^. When the amount of Fe^3+^ increased in FPB and VFPB2, Fe^2+^ 2p peaks appeared at 708.4 and 721.4 eV and Fe^3+^ 2p peaks appeared at 709.8 and 723.2 eV, which are ascribed to 2p_3/2_ and 2p_1/2_ orbitals, respectively. The V 2p XPS spectra of VPB, VFPB1, VFPB2, and FPB are shown in Figure 2b. The V 2p spectra showed two peaks at ~517 and 525 eV, which correspond to V 2p_2/3_ and V 2p_1/2_ orbitals and can be fitted to the peaks at 516.3/523.8 and 517.3/524.8 eV and were ascribed to V^4+^ and V^5+^ states, respectively. A V^3+^ peak was not observed, indicating that it was oxidized to V^4+^ during the synthesis. A small V^5+^ peak was observed in VPB and VFPB1, which can be attributed to the oxidation of V^4+^ on the surface. The ratio of V^5+^ to V^4+^ peak intensities in VFPB2 indicates that V^4+^ is unstable in the presence of Fe^3+^. In addition, this oxidation of V^3+^ and reduction of Fe^3+^ were indicated by a color change from yellow-blue to blue, as shown in Figure 2c. The full scan of XPS spectra for all samples was also recorded and illustrated in Figure S3. The percentage of each element is analyzed and summarized in Figure 2d. The CN:Fe ratio is larger than 7 for VPB and VFPB1, which is ascribed to Fe-ion defects in the lattice or the addition of V ions in the complex, resulting in different electrochemical properties [37]. In contrast, the CN:Fe ratio in VFPB2 and FPB is approximately 3.7–4.8, which is consistent with those reported for several Prussian blue analogs [38,43]. K ions were also detected owing to the use of excess K_3_Fe(CN)_6_ during synthesis, which is in good agreement with previous reports on PBA synthesis [35,44,45]. Therefore, the molecular formula of the final compound was established as K_x_(VO)_y_Fe_z_(CN)_6_.

**Figure 3:**
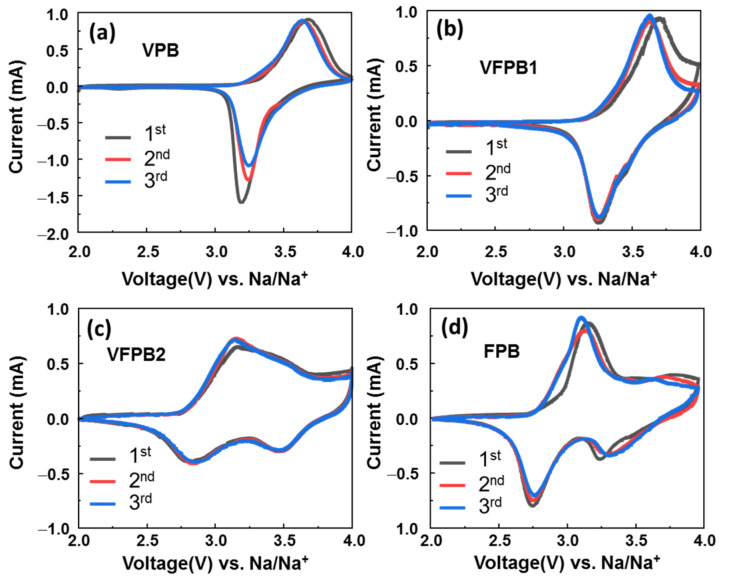
Cyclic voltammetry (CV) profiles of the first three cycles of (**a**) VPB, (**b**) VFPB1, (**c**) VFPB2, and (**d**) FPB cathodes.

Next, all samples were employed as cathodes in half-cell SIBs to investigate their electrochemical properties. Figure 3 shows the CV plots of VPB, VFPB1, VFPB2, and FPB electrodes in the voltage range of 2.0–4.0 V vs. Na/Na^+^ at a scan rate of 0.1 mV s^−1^. The redox reactions of VPB and VFPB1 electrodes were identified by coupled peaks at 3.26/3.65 V vs. Na/Na^+^, which are attributed to the reduction/oxidation of [Fe(CN6)]^3−^/[Fe(CN6)]^4−^ [29,46]. The VFPB1 electrode showed more stable cycling after the first cycle compared to that of pure VPB. The VFPB2 and FPB electrodes exhibited coupled redox peaks at 2.8/3.1 and 3.26/3.65 V vs. Na/Na^+^, which are ascribed to the redox reaction of Fe^3+^/Fe^2+^ ions [29]. Based on previous reports, the first coupled peak at 2.8/3.1 V belonged to the redox reaction of high-spin Fe^3+^/Fe^2+^, whereas the redox coupled peaks at 3.26/3.65 V were attributed to low-spin Fe^3+^/Fe^2+^ [36,47,48]. The VFPB2 electrode has a higher reduction potential of ~3.5 V at 50 mA g^−1^; however, this peak shifted to 3.2 V, indicating the unstable structure of VFPB2. The analysis of the CV curves revealed that a certain amount of V in the lattice structure of FPB (i.e., in VFPB1) passivated the high-spin Fe^2+^ and, consequently, activated only low-spin Fe^2+^, resulting in a higher redox potential than that in samples without V.

**Figure 4:**
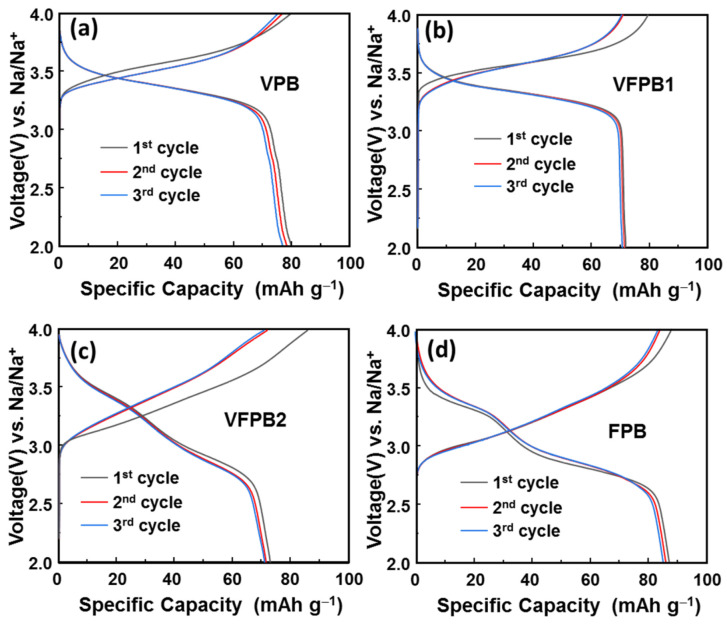
Initial voltage profiles of (**a**) VPB, (**b**) VFPB1, (**c**) VFPB2, and (**d**) FPB cathodes.

Figure 4 shows the initial voltage profiles of VPB, VFPB1, VFPB2, and FPB electrodes in the voltage range of 2.0–4.0 V vs. Na/Na^+^. The voltage profiles of electrodes with high and low V concentrations were different. During discharging, VPB and VFPB1 demonstrated ~3.3 V, whereas VFPB2 and FPB exhibited two voltage platforms at approximately 3.4–3.5 and 2.7 V. These discharge platforms corresponded to the cathode reduction potentials. Thus, the use of V in PBAs passivates high-spin Fe^3+^/Fe^2+^ and retains only low-spin Fe^2+^ ions, thus enhancing the voltage profiles of the batteries. The incorporation of V in PBA results in a capacity-voltage profile trade-off where the capacity is slightly reduced while the voltage platform is increased. However, a mixed state of Fe^2+^, Fe^3+^, and V^4+^ exists in the PBA lattices of the VFPB2 electrode, which makes its electronic structure unstable and induces a faster capacity decay than those in the other electrodes.

**Figure 5:**
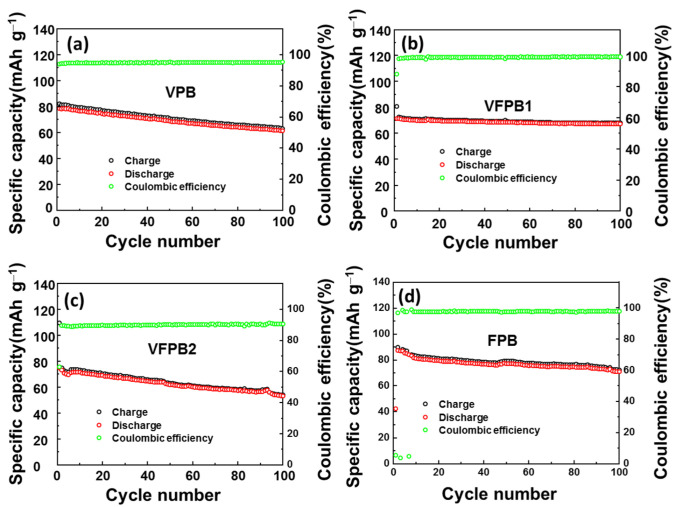
Cycling performances of (**a**) VPB, (**b**) VFPB1, (**c**) VFPB2, and (**d**) FPB cathodes at 50 mA g^−1^ over 100 cycles.

Subsequently, a cycling test was conducted at a current rate of 50 mA g^−1^ to test the performance of Fe-V-incorporated PBAs, as shown in Figure 5. After 100 cycles, the VPB electrodes showed a gradual capacity degradation from ~80 to 60 mAh g^−1^, whereas that of the VFPB2 electrodes degraded from ~110 to 54 mAh g^−1^. In addition, the FPB electrode exhibited the highest initial capacity of 90 mAh g^−1^, which reduced gradually to ~70 mAh g^−1^ after 100 cycles. Even though the VFPB1 electrode started with a low capacity, it showed a stable reversible capacity of ~70 mAh g^−1^. The capacity retention was ~82.5% of the initial capacity (~80 mAh g^−1^). Next, the rate performance was investigated to test the performance of the prepared cathode materials, as shown in Figure S4. The VPB, VFPB1, VFPB2, and FPB electrodes showed a low capacity of 10–15 mAh g^−1^ at a current rate of 1.0 A g^−1^. When the current changed from 1 to 50 mA g^−1^, the VPB electrode showed a low-capacity restoration of ~88%, whereas the VFPB1, VFPB2, and FPB electrodes demonstrated a high-capacity restoration of ~98%. The long-term stability test of the VFPB1 cell was also conducted by applying a current density of 50 mA g^−1^ for 100 cycles, 500 mA g^−1^ for 10,000 cycles, and 50 mA g^−1^ for 35 cycles, as shown in Figure S5. It shows that the capacity of the VFPB1 cell can be restored to 54 mAh g^−1^ even after 10,000 cycles, which is about 77% of its initial capacity. Therefore, it can be concluded that the VFPB1 cathode is very stable in the long-term cycling of SIBs.

**Figure 6:**
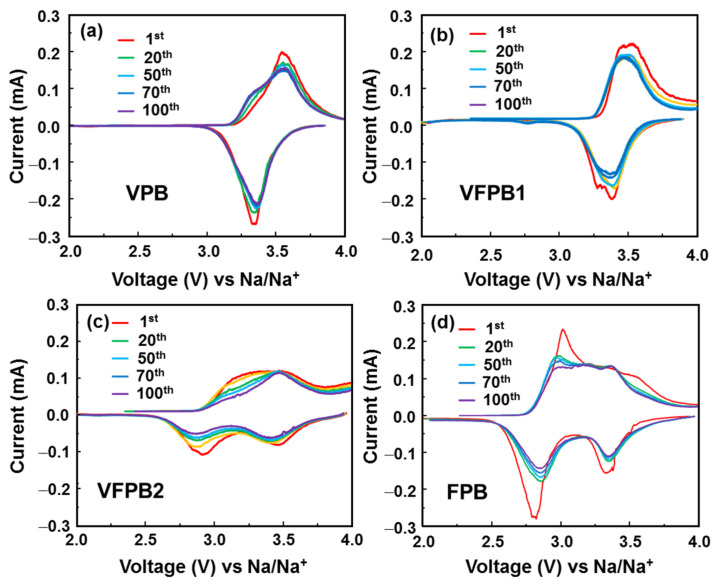
DCP plots during charge/discharge process at various cycles for (**a**) VPB, (**b**) VFPB1, (**c**) VFPB2, and (**d**) FPB cathodes.

In addition, the differential capacity plots (DCPs) were drawn to study the electrochemical behaviors of the electrodes at the 1st, 20th, 50th, 70th, and 100th cycles, as illustrated in Figure 6. According to previous reports, the insertion/extraction of Li ions into VPB could change the electrochemical properties of the Prussian blue material [49]. The DCP results of the VPB material showed a change in the redox peaks, where the VPB electrode revealed a change from a single redox peak to two redox peaks. This was related to the low spin of Fe ions and the low/high spin of Fe ions. In this work, the slight changes observed in the DCP peaks of the VPB, VFPB1, and FPB electrodes indicate the stability and electrochemical performance of the unified structure. However, the mixed state of VPB and FPB in the VFPB2 electrode resulted in high-capacity degradation, which indicates a decrease in the high-spin Fe^2+^/Fe^3+^ redox peak intensity at 2.8/3.1 V. Furthermore, the FPB cathode showed a gradual decrease in the high- and low-spin Fe^2+^ redox peak intensities. VPB and VFPB1 electrodes showed small changes in the DCP curves, indicating highly stable electrochemical performances. The VPB cathode exhibited a shoulder peak at ~3.3 V after the first cycle, which suggests an unstable structure. Fe in VPB and VFPB1 electrodes is in the Fe^2+^ state, but the amount of Fe is higher than that of V in VFPB1. As mentioned earlier, the electrochemical redox potential of Fe^3+^/Fe^2+^ is higher than that of V^3+^/VO^2+^; therefore, the additional FeCl_3_ led to the rapid oxidation of V^3+^. The fast oxidation of V^3+^ facilitates the rapid formation of VPB, resulting in structure as well as cyclic stability. However, in the VFPB2 electrode, the higher amount of FeCl_3_ led to the coexistence of Fe^3+^ and Fe^2+^ (V:Fe = 1:1.63). This mixed structure comprises V^4+^, Fe^2+^, and Fe^3+^; therefore, it may not be stable. Thus, the stability of the VFPB2 cathode was worse than that of the FPB cathode. In summary, an additional but small amount of FeCl_3_ was introduced in VFPB1 (V:Fe = 1:1.16) for the rapid formation of the VPB structure, which reduced the V^4+^ and Fe^2+^ defects and stabilized their structures, consequently imparting better electrochemical stability to the cathode material.

**Figure 7:**
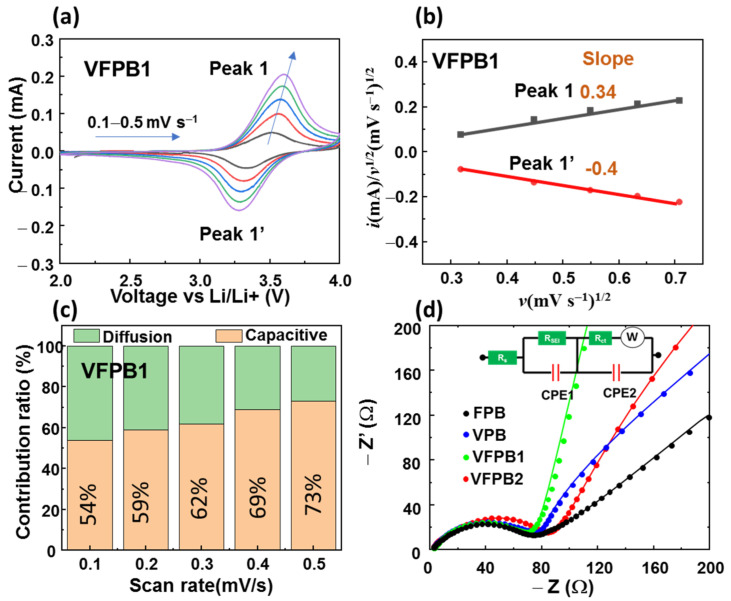
(**a**) CV curves; (**b**) fitted lines of *i*/*v*^1/2^ vs. *v*^1/2^ at different scan-rate voltages (*v*) from 0.1–0.5 mV s^−1^; (**c**) capacitance- and diffusion-controlled contribution ratios of the currents of VFPB1 cathodes; and (**d**) Nyquist plots for impedance measurement of FPB, VPB, VFPB1, and VFPB2 cathodes with inset of equivalent circuit.

Subsequently, a kinetic analysis was conducted via sweep rate voltammetry to further understand the behavior of the VFPB1 cathode. Figure 7a shows the CV curves of VFPB1 in the scan-rate range of 0.1–0.5 mV s^−1^. The contributions of diffusion- and capacitive-controlled processes can be determined via Dunn’s method. The measured current intensity of the cell can be expressed as *i* = *k*_1_*v + k*_2_*v*^1/2^, where *k*_1_*v* and *k*_2_*v*^1/2^ are the contributions from capacitance and diffusion to the current, *v* is the voltage scan rate, and *k*_1_ and *k*_2_ are the constants. The plots of *i*/*v*^1/2^ and *v*^1/2^ were drawn and fitted to determine the constants, as shown in Figure 7b. The contributions of diffusion and capacitive behaviors were calculated per unit scan rate, as shown in Figure 7c. The VFPB1 cathode showed an equivalent contribution of capacitance and diffusion at 0.1 mV s^−1^ with ratios of ~54 and 46%, respectively. As the scan rate increased, the capacitive contribution in the VFPB1 cathode increased rapidly to 73% at 0.5 mV s^−1^. Therefore, the incorporation of V in FPB enhanced the capacitive contribution of the Prussian blue cathode with increasing scan rate, which could be attributed to the consolidation of two redox reactions into a single redox reaction. The impedance spectra of FPB, VPB, VFPB1, and VFPB2 were also conducted, as shown in Figure 7d. Four samples show a low charge transfer resistance (R_ct_) of ~80 Ω. Specifically, the FPB and VPB show similar R_ct_ of ~73 Ω. Meanwhile, the VFPB1 cathode has the lowest R_ct_ of ~70 Ω and VFPB2 has the highest R_ct_ of ~94 Ω. According to the EIS literature, the semicircle at high frequency is related to the internal resistance of the electrode, while the curve at low frequency is related to diffusion layer resistance [50]. In this work, the Nyquist plot of the VFPB1 cathode was more linear and showed higher growth than that of the VFPB2 cathode, which indicates that the VPFB1 cathode has lower diffusion layer resistance than the VFPB2 cathode. Therefore, the sodium ions can be easily diffused into the VFPB1 structure, leading to an improvement in electrochemical properties.

Table 1 shows a comparison of recently reported Prussian blue analog-based cathodes and the VFPB1 one from this study. The ideal cathode for alkaline-ion batteries (Li, Na, K) should have a simple synthetic method, low cost, high specific capacity, a high voltage platform, high stability or cycling stability, and high rate performance. However, obtaining all the above factors is difficult, especially the rate performance. For example, at high current densities, the capacities could be significantly reduced based on the active materials, loading methods, or porosity [51,52]. Moreover, intrinsic PBAs possess poor electrical conductivity; therefore, modification is essential to improve their electrochemical properties, which could include the modification of the structure or composition using high-valence-state transition metals (V, Mn) [23]. Pure PBAs of sodium and iron ferrocyanides exhibit a high capacity at a low current of ~10/20/25 mA g^−1^ and two redox potentials. The incorporation of metal ions such as V, Mn, Ni, and Cu into PBAs enhances their electrochemical performance. Previous studies have shown that the use of V ions would result in a single redox potential. In this study, the Fe-V-incorporated PBA in the VFPB1 electrode exhibited a coupled redox potential of 3.26/3.65 V at a scan rate of 0.1 mV s^−1^. This could facilitate stable SIB voltage profiles for their commercialization. The reversible capacity of 70 mAh g^−1^ at a rate of 50 mA g^−1^ is comparable to that of other modified PBAs. However, further improvements in rate performances and capacities are required, which could be resolved via surface engineering or combination with derivative carbons such as carbon nanotubes or graphene.

## 4. Conclusions

We explored vanadium Prussian green and Fe-V-incorporated Prussian blue as cathode materials for SIBs. The XRD, XPS, and TEM measurements revealed that the presence of V^3+^ induced the reduction of [Fe(CN)_6_]^3−^ to [Fe(CN)_6_]^4−^ and controlled its own oxidation to V^4+^. The low crystallinity of the as-prepared samples was attributed to the formation of small nanoparticles in the size range of 10–30 nm. VFPB1 as the Na-ion cathode showed good electrochemical performance and a stable voltage platform of 3.26–3.65 V. The cycling test revealed that VFPB1 exhibited a high and stable capacity of ~70 mAh g^−1^ and high capacitance recovery of 96% when the current rate decreased from 1000 to 50 mA g^−1^. Even though a high, stable voltage was achieved, further improvements in rate performance and capacity are required before it could be used for practical applications.

## Figures and Tables

**Table 1:** Comparison of the performances of Prussian blue analog-based cathode materials for sodium-ion batteries.

Materials	Redox Couple Potential (V) vs. Na/Na^+^	Current Rate (mA g^−1^)	Specific Capacity (mAh g^−1^)	Ref.
NaFe[Fe(CN)_6_]	2.6/2.9 and 3.3/3.6	10	118.2	[46]
Fe_4_[Fe(CN)_6_]_3_	2.8/3.0 and 3.38/3.53	20	146	[30]
Na_2_V[Fe(CN)_6_]	3.2/3.6	~2.5 (C/20)	50	[37]
H_2_O-removed Na_2_MnFe(CN)_6_	-	100	150	[53]
Na_4_Fe(CN)_6_	2.6/3.1 and 3.3/3.7	25	170	[54]
Na_0.86_Ti_0.73_[Fe(CN)_6_]	2.5/2.6 and 3.5/3.6	~4 (C/20)	74	[55]
Ni rich PBA	2.5/3.0 and 3.2/3.6	10	120	[56]
R-Na_1.92_Fe[Fe(CN)_6_	3.00/3.11 and 3.29/3.30	10	120	[57]
Na_4_Fe(CN)_6_/C	3.25/3.5	9 (~0.1 C)	90	[22]
Na_2−x_Fe[Fe(CN)_6_]	2.82/3.03 and 3.31/3.45	10	120	[36]
Mn/Ni PBA	3.1/3.4 and 3.5/3.6	0.1 C	100	[35]
Na_1.76_Ni_0.12_Mn_0.88_[Fe(CN)_6_]_0.98_	2.9/3.3	100	80	[58]
NaVHCF	3.2/3.6	~20 (0.25 C)	70	[38]
VFPB1	3.26/3.65	50	70	This work
		100	60	

## Data Availability

The data presented in this study are available on request from the corresponding author.

## Supplementary Materials

The following supporting information can be downloaded at https://www.mdpi.com/article/10.3390/mi14030521/s1, Figure S1: Scanning electron microscopy images of (a) VPB, (b) VFPB1, (c) VFPB2, and (d) FPB materials. Figure S2: Transmission electron microscopy images with inset selected area electron diffraction patterns of (a) VPB, (b) VFPB1, (c) VFPB2, and (d) FPB materials. Figure S3: X-ray photoelectron spectroscopy spectra of VPB, VFPB1, VFPB2, and FPB materials. Figure S4: Rate performance of (a) VPB, (b) VFPB1, (c) VFPB2, and (d) FPB cathodes at currents of 50, 100, 200, 500, and 1000 mA g^−1^. Figure S5. (a) Long-term stability test of VFPB1 cell for 100 cycles at 50 mA g^−1^, for 10,000 cycles at 500 mA g^−1^, and for 35 cycles at 50 mA g^−1^; (b) cycling performance at 50 mA g^−1^ after 10,000 cycles.
