# Supplementary material for: Iron-Vanadium Incorporated Ferrocyanides as Potential Cathode Materials for Application in Sodium-Ion Batteries
Source: Micromachines (Basel). 2023 Feb 23;14(3):521. doi: 10.3390/mi14030521 (PMC10059089; doi:10.3390/mi14030521)
Supplement: Supplementary file 1 [file micromachines-14-00521-s001.zip › micromachines-2231930-supplementary.pdf]

SUPPORTING INFORMATION

Article

Iron-Vanadium Incorporated Ferrocyanides as Potential Cathode Materials for Application in Sodium-Ion Batteries

Thang Phan Nguyen and Il Tae Kim*

Department of Chemical and Biological Engineering, Gachon University, Seongnam-si 13120, Gyeonggi-do, Republic of Korea; phanthang87@gmail.com
* Correspondence: itkim@gachon.ac.kr

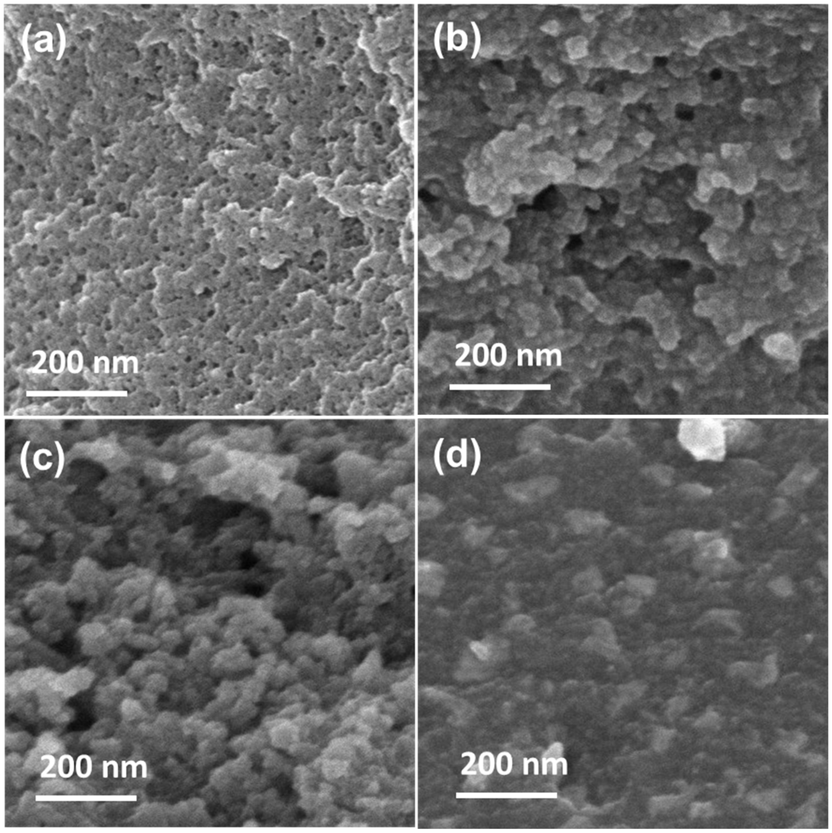

Figure S1. Scanning electron microscopy images of (a) VPB, (b) VFPB1, (c) VFPB2, and (d) FPB materials.

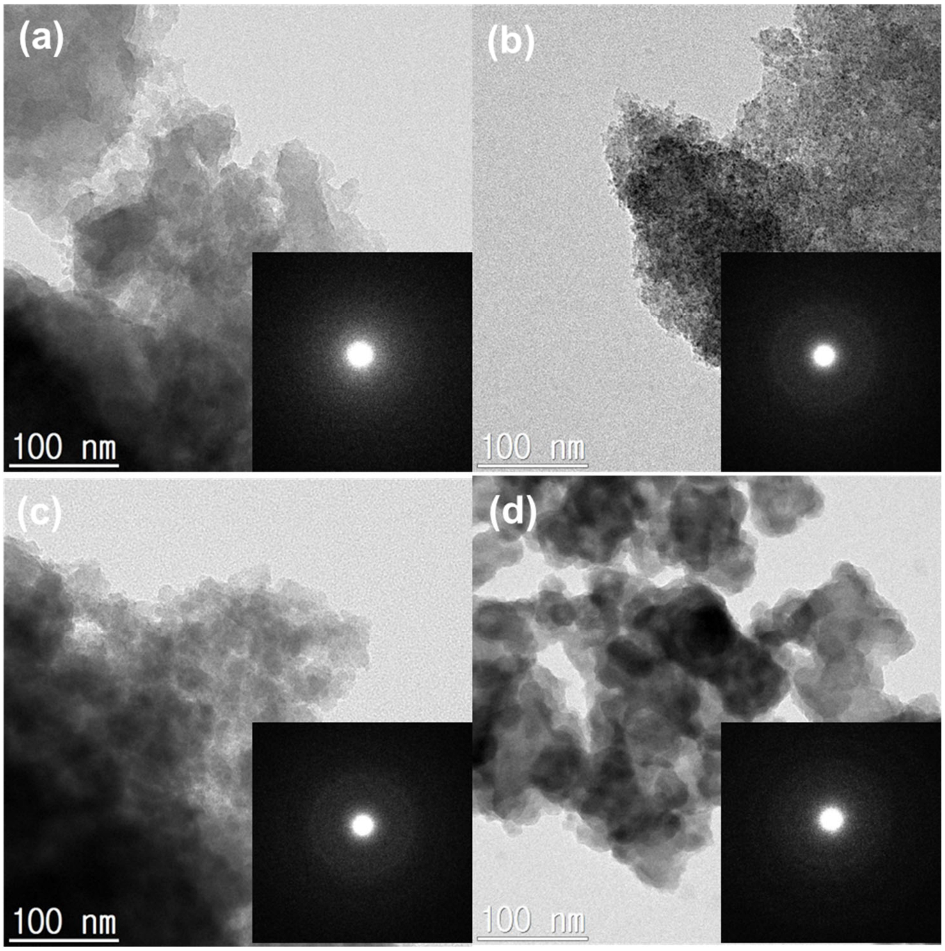

Figure S2. Transmission electron microscopy images with inset selected area electron diffraction patterns of (a) VPB, (b) VFPB1, (c) VFPB2, and (d) FPB materials.

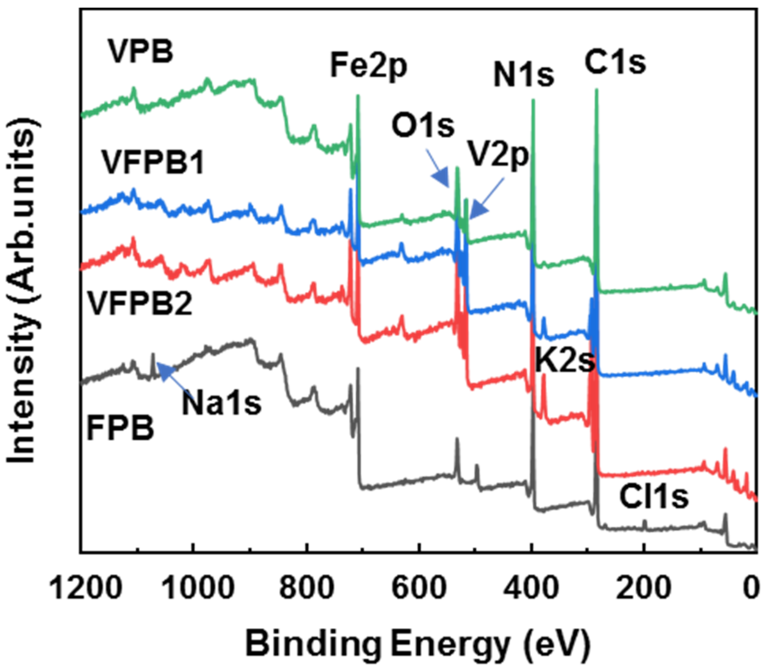

Figure S3. X-ray photoelectron spectroscopy spectra of VPB, VFPB1, VFPB2 and FPB materials.

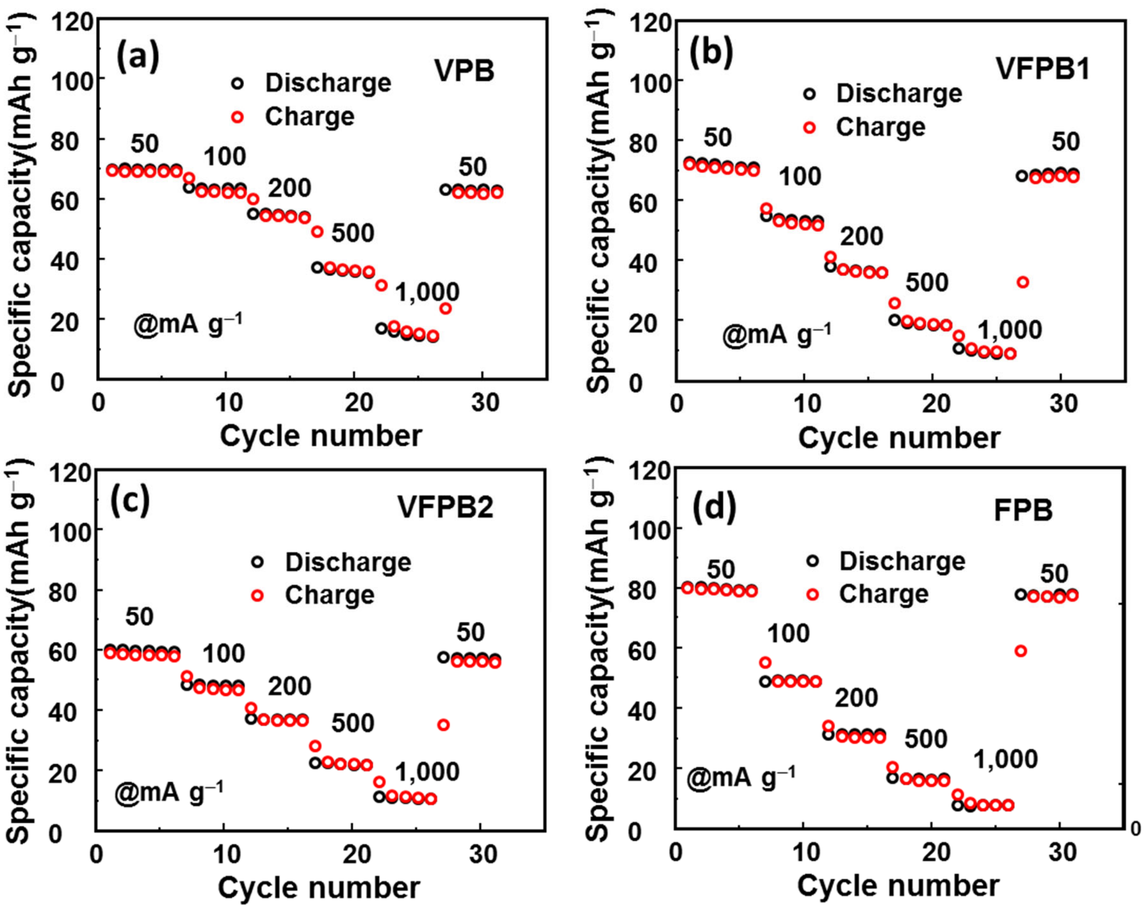

Figure S4. Rate performance of (a) VPB, (b) VFPB1, (c) VFPB2, and (d) FPB cathodes at currents of 50, 100, 200, 500, and 1000 mA g^{-1}.

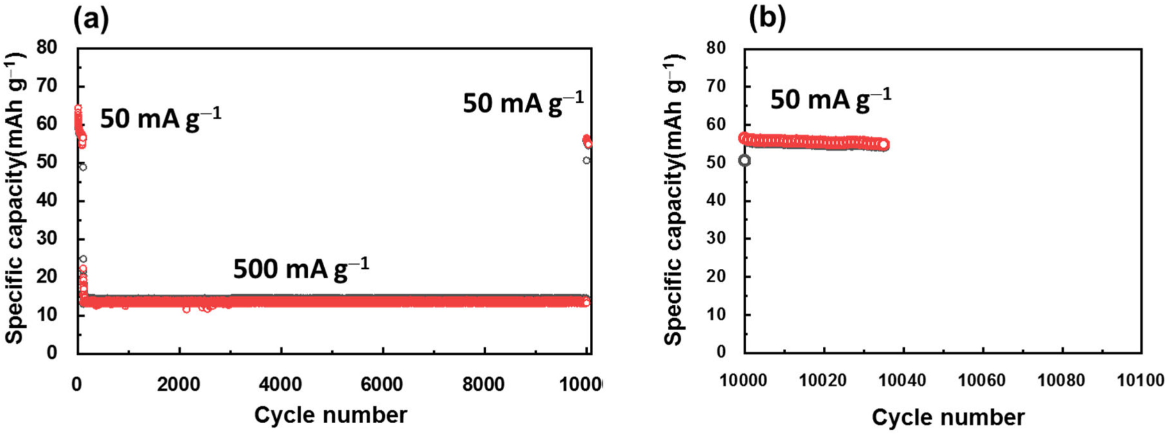

Figure S5. (a) Long-term stability test of VFPB1 cell for 100 cycles at 50 mA g^{-1}, for 10,000 cycles at 500 mA g^{-1}, and again for 35 cycles at 50 mA g^{-1}; (b) cycling performance at 50 mA g^{-1} after 10,000 cycles.
